# Benign Brenner tumor of the ovary: two-dimensional and contrast-enhanced ultrasound features—a retrospective study from a single center

**DOI:** 10.3389/fonc.2024.1337806

**Published:** 2024-03-08

**Authors:** Mei Chen, Shusheng Liao, Yong Cao, Meiya Mao, Xiupeng Jia, Shengmin Zhang, Youfeng Xu

**Affiliations:** ^1^ Department of Ultrasonography, Ningbo First Hospital, Ningbo, Zhejiang, China; ^2^ Department of Ultrasound, The First Affiliated Hospital of Wenzhou Medical University, Wenzhou, Zhejiang, China; ^3^ Department of Gynecology, Ningbo First Hospital, Ningbo, Zhejiang, China; ^4^ Department of Histology, Ningbo First Hospital, Ningbo, Zhejiang, China

**Keywords:** Brenner tumor, pathology, benign, diagnosis, surgery, ultrasound

## Abstract

**Objective:**

Benign Brenner tumor (BBT) is a rare ovarian tumor, and there are few discrete reports about its manifestation in an ultrasound. This study sought to investigate the two-dimensional (2D) and contrast-enhanced ultrasound (CEUS) features of this entity.

**Methods:**

This is a retrospective single-center study. The clinical manifestations, laboratory examination, and ultrasound data of 25 female patients with BBT were confirmed by pathology when they underwent 2D and/or CEUS examination at Ningbo First Hospital from January 2012 to June 2023. The ultrasound findings of the patients were analyzed using the terminology of the International Organization for the Analysis of Ovarian Tumor and were read by two senior sonographers who reached an agreement.

**Results:**

Among the all 25 patients, most of them were unilateral, and only one patient was bilateral. Thus, 26 lesions were found: 44.0% (11/25) were in the left and 52.0% (13/25) were in the right. Moreover, 53.84% (14/26) were solid lesions, 15.38% (4/26) were mixed lesions, and 26.92% (7/26) were cystic lesions. Among the solid-type patients, 42.85% (6/14) of the cases were with calcification. Upon laboratory examination, 12.0% (3/25) of the patients had high carbohydrate antigen 125 (CA-125) level, and 19.04% (4/21) of the patients had an elevated carbohydrate antigen724 (CA-724) level in the serum tumor markers. In the hormone test, 14.28% (3/21) were found to have a high postmenopausal estrogen level and 14.28%(3/21) were found to have a high level of follicle-stimulating hormone (FSH). One patient with complex manifestations and three with solid manifestations were examined by CEUS to observe the microcirculation perfusion of the tumor. One with solid and cystic separation was rapidly hyperenhanced and cleared, and the filling subsided faster than the uterus. The postoperative pathological diagnosis was benign Brenner tumor with mucinous cystadenoma. The other three cases were solid adnexal lesions, which showed isoenhancement on CEUS and disappeared slowly, synchronizing with the uterus. The CEUS results were considered as benign tumors and confirmed by pathology.

**Conclusions:**

BBT can show ovarian cystic, mixed cystic and solid type, and solid echo in 2D ultrasound. Unilateral ovarian fibrosis with punctate calcification is an important feature of BBT in 2D ultrasound. However, for solid adnexal masses and mixed cystic and solid masses with unclear diagnosis, if CEUS shows isoenhancement or hyperenhancement, the possibility of BBT cannot be excluded.

## Introduction

MacNaughton-Jones was the first to report on Brenner tumors (BT) in 1898, and then Fritz Brenner described the tumor that bears his name in 1907 (1). It is a rare ovarian tumor derived from the transitional epithelium of the urinary tract and accounts for approximately 2%–3% of epithelial tumors in the ovary (2). Ovarian transitional cell tumor has been replaced by BT according to the latest classification of ovarian cancer by the World Health Organization (3). BT has three subtypes: benign Brenner tumor (BBT), which is by far the most common; borderline, also known as dysplastic BT; and malignant. Borderline and malignant BTs are extremely rare, accounting for less than 10% of BTs (4), and tend to occur in older patients (5). Most of the benign tumors are between 5 and 6 cm in diameter, and a few cases are more than 10 cm (6). Borderline and malignant BTs can reach a maximum diameter of 30 cm (7). Most patients are asymptomatic, but some may experience abdominal pain, vaginal bleeding due to estrogen activity, urinary retention, ascites, or pseudo-Meigs syndrome (8). BTs can occur at any age, but mostly at the ages of 50–70 years, and are often detected incidentally during routine physical and/or ultrasound examination (9). Preoperative ultrasound examination is very important for the treatment and differentiation of benign and malignant ovarian lesions. However, although the International Ovarian Tumor Analysis (IOTA) group has established the imaging criteria for ovarian cancer identification, the ultrasound appearance of BT overlaps with the appearance of a typical ovarian cancer, leading to image bias (10, 11). BTs are usually reported in individual cases. Some previous case reports have mentioned that the presence of calcification within the lesion is the imaging feature of this tumor (12). An accurate understanding of the ultrasound images of BBT before a surgery can reduce the patients’ fear of ovarian cancer diagnosis, and appropriate surgical methods can prevent overtreatment. To date, the integrated ultrasound characteristics of BTs are still lacking. Thus, this study retrospectively analyzed the clinical data and ultrasound findings of 25 patients with BBT and explored the ultrasound features of this entity to provide evidence for clinical diagnosis and treatment.

## Materials and methods

### Study population

This was a retrospective study conducted in a single center, namely, Ningbo First Hospital. Ethical approval for this study was obtained, and all the patients provided a written informed consent. A computerized search of the pathology records of this institution from January 2012 to June 2023 was performed. This resulted in the identification of 26 patients with a histological diagnosis of BBT. A second search was then conducted on the image archive and communication system to identify patients who underwent 2D and/or contrast-enhanced ultrasound (CEUS) examination before surgery. One was excluded due to the presence of the lesion at imaging the actual vagina, which was diagnosed as BBT by colposcopy. Therefore, a total of 25 patients were identified, and one of them had bilateral lesions. Finally, all the 25 patients with 26 lesions had 2D examination data, and four of them had the results of 2D and CEUS.

### Materials

A retrospective analysis was conducted on 25 patients with pathologically confirmed BBT and in whom 2D and/or CEUS examinations were performed in our hospital from January 2012 to June 2023. The patients aged from 32 to 77 years, with an average age of 61.37 ± 9.67 years. The clinical data included age at diagnosis, pre- or postmenopausal status, symptoms, presence of ascites, and the clinical stage [the IOTA consensus as described by Timmerman et al. (11)].

Laboratory examination such as the level of carbohydrate antigen 125 (CA-125), carbohydrate antigen 199 (CA-199), carbohydrate antigen 153 (CA-153), carbohydrate antigen 724 (CA-724), alpha-fetoprotein (AFP), estrogen levels (E2), follicle-stimulating hormone (FSH), and prolactin (PRL) in the blood, symptoms, and presence of ascites were according to the IOTA consensus described by Timmerman et al. (11). The clinical data, laboratory examination, and 2D ultrasound image of the 25 patients with 26 lesions as well as the CEUS image of four patients were analyzed. The type of surgery (laparotomy or laparoscopy) and the extent of surgical resection, such as unilateral oophorectomy or more extensive total hysterectomy, or even bilateral salpingo-oophorectomy, were recorded and analyzed. The postoperative pathology of each patient was also recorded in detail. The clinical and ultrasound data were imported into Excel (Microsoft Office Excel 2010) and then analyzed. All patients signed the informed consent form, and the study was approved by the Medical Ethics Committee of Ningbo First Hospital (no. 2023RS125).

### Sonographic examination

GE E8, PHILIPS iU22, TOSHIBA Aplio500, and other Color Doppler ultrasound diagnostic instruments were used. For a comprehensive evaluation, transvaginal probe frequency of 5–9 MHz, transabdominal probe frequency of 3.5–5.0MHz, routine transvaginal scanning, and partial transabdominal ultrasonography were adopted. A comprehensive analysis was conducted according to the tumor’s size, boundary, shape, location, internal echo, and blood flow as well as the presence of calcification, fibroids, polyps, and thoracoabdominal effusion. The ultrasound contrast agent in this study was SonoVue (Bracco, Milan, Italy). A 2.4-mL bolus of SonoVue was injected intravenously through the cubital vein, followed by a 5-mL saline flush to ensure that no residual contrast agent remained in the intravenous catheter. Imaging started immediately after injection and lasted for 2 to 4 min. The pathological diagnosis was the gold standard.

### Statistical method

SPSS 24.0 for Windows (SPSS, Chicago, IL, USA) was used for statistical analysis and chart drawing. Qualitative variables are represented using absolute values and percentages, and continuous variables are represented using mean ± standard deviation (SD).

## Results

### Clinical history

There were 26 nodules in 25 patients, of which one had bilateral nodules and the rest had unilateral nodules. Of the 25 patients, 52.0% (13/25) had BT in the right ovary and 44.0% (11/25) in the left ovary; 36.0% (9/25) showed symptoms of abdominal pain, abdominal distension, and vaginal bleeding; 64.0% (16/25) showed no symptoms and had normal physical examination results; 8.0% (2/25) had normal menstruation; and 92.0% (23/25) were menopausal.

### Laboratory examination

In the tumor marker test, 12.0% (3/25) of the patients had a high CA-125 level, which was 69.6 U/mL, with the normal level being 30 U/mL. Furthermore, 19.04% (4/21) of the patients had an elevated CA-724 level, which was 12.02 U/mL, with the normal level being 7 U/mL. Moreover, 4% (1/25) of the patients had a high CA-199 level, which was 29.9 U/mL, with the normal level being 25 U/mL. No obvious abnormality was observed in CA-153, alpha-fetoprotein (AFP), carcinoembryonic antigen (CEA), human chorionic gonadotropin (HCG), and CA-153 in the remaining patients who underwent the test in blood. Of the 25 patients, 21 underwent reproductive hormone examination, of whom 14.28% (3/21) were found to have a high postmenopausal estrogen level, 14.28% (3/21) were found to have a high FSH level, and 4.76% (1/21) were found to have a high PRL level. No obvious abnormality was observed in LH, P, and T of 21 patients who underwent hormone examination (**Table 1**).

**Table 1:** Clinical characteristics and surgical management of the 25 patients with benign Brenner tumor (BBT).

Parameters		BBT patients (*n* = 25)
Age at surgery (years)		63 (32–77)
Serum CA125 level (U/mL) Reference range(0–30)	<30	88.0% (22/25)
	>30	12.0% (3/25)
Serum CA-724 level (U/mL) Reference range (0–7)	<7	80.95% (17/21)
	>7	19.04% (4/21)
Serum CA-153 level (U/mL) Reference range (0–25)	<25	100% (23/23)
	>25	
AFP level (ng/mL) Reference range (0–25)	<25	100% (25/25)
	>25	
CEA level (U/mL) Reference range (0–10)	<10	100% (25/25)
	>10	
Serum CA-199 level (U/mL) Reference range (0–25)	<37	95.23% (20/21)
	>37	4.76% (1/21)
Estrogen levels (IU/L)	Normal	90.47% (19/21)
	Abnormal	9.53% (2/21)
Follicle-stimulating hormone (FSH)	Normal	90.47% (19/21)
	Abnormal	9.53% (2/21)
Prolactin (PRL)	Normal	95.0% (19/20)
	Abnormal	5.0% (1/20)
Menstruation	Premenopausal	8.0% (2/25)
	Postmenopausal	92.0% (23/25)
Symptomatology	Asymptomatic	64.0% (16/25)
	Pelvic pain	36.0% (9/25)
Surgery	Salpingo-oophorectomy laparoscopy	24.0% (6/25)
	Total laparoscopic hysterectomy	72.0% (18/25)
	Total abdominal hysterectomy	4.0% (1/25)
Pathology	Pure Brenner	72.0% (18/25)
	Brenner + mucinous cystadenoma	12.0% (3/25)
	Brenner + serous cystadenoma	4.0% (1/25)
	Brenner + seromucinous cystadenoma	4.0% (1/25)
	Brenner + ovarian fibroma	4.0% (1/25)
	Brenner + endometrioid cyst	4.0% (1/25)

Values are expressed as median (range) or n (%).

### Ultrasound characteristics

**Figure 1:**
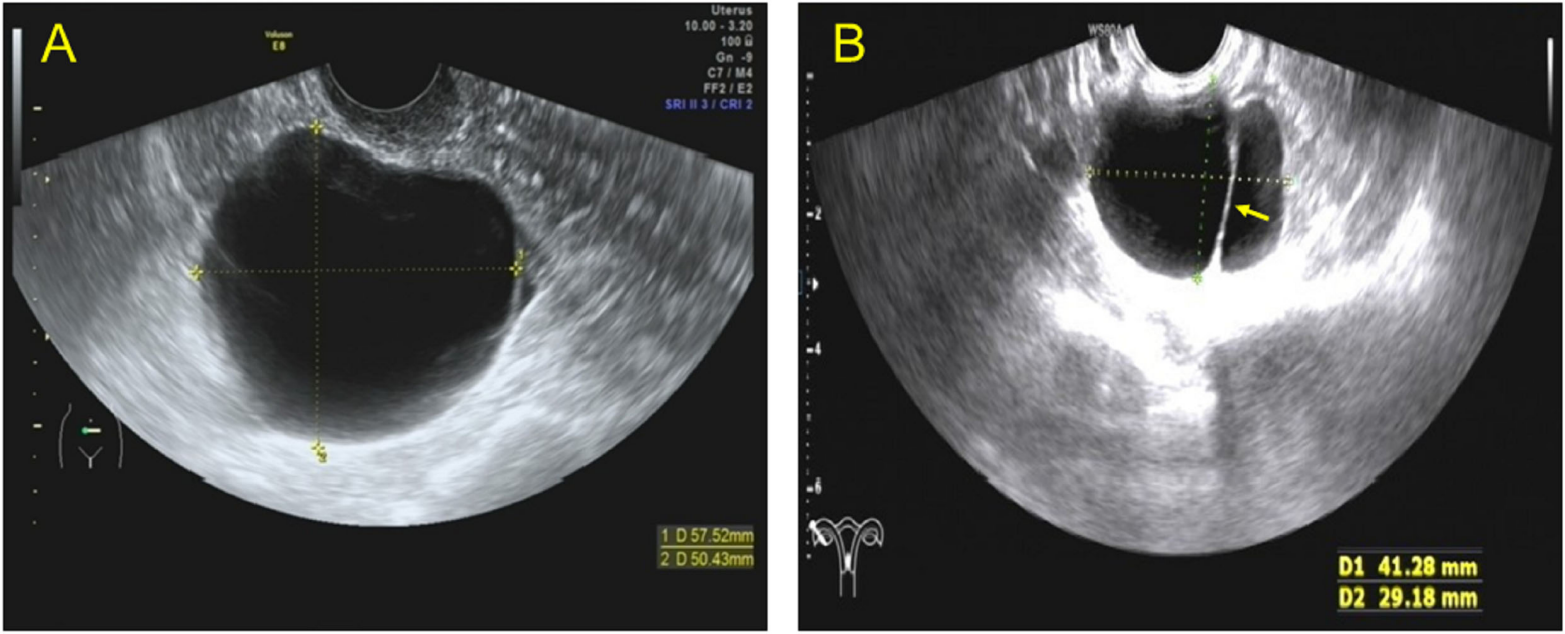
Transvaginal ultrasound images showing the cystic type lesions of benign Brenner tumor. **(A)** Transvaginal ultrasound (TVU) showing a case of unilocular lesions with thin walls. **(B)** TVU showing a case of cystic lesions with thin separation in the right ovary.

Only one patient was pathologically confirmed to have bilateral BBTs, and 96%(24/25) of patients were pathologically confirmed to have unilateral BBTs with the following ultrasound characteristics: (1) site: left in 44.0% (11/25), right in 52.0% (13/25); (2) size (diameter of the lesion): 1.7–16.0 cm; (3) shape and boundary: round, quasi-round, or irregular, with clear boundaries; (4) internal echo and blood flow: cystic lesions in 30.76% (8/26), mixed cystic and solid lesions in 15.38% (4/26), and solid lesions in 53.84% (14/26). Among the eight cases of cystic lesions, three (37.5%) were unilocular with thin walls (**Figure 1A**). Thin hyperechoic septa were detected in two (**Figure 1B**). Through color Doppler flow imaging (CDFI), blood flow distribution was observed in the cyst wall.

**Figure 2:**
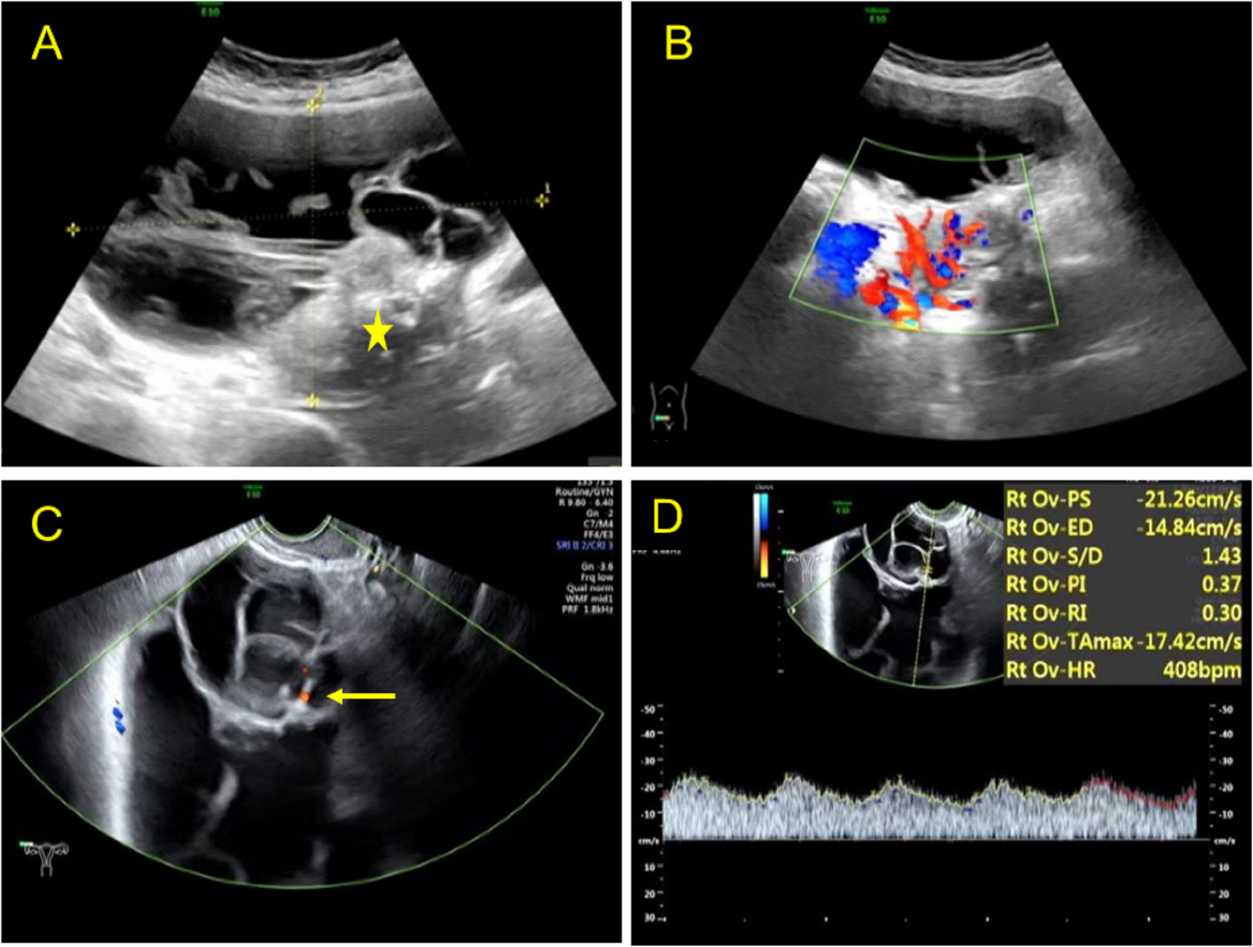
Ultrasound images of a benign Brenner tumor patient of the mixed cystic and solid type. **(A)** Transabdominal sonography showing a giant mixed cystic and solid (asterisk) lesion in the right lower abdomen with a diameter of 15.1 × 9.8 cm. **(B)** Color Doppler imaging revealed an abundant blood flow in the solid part of the lesion. **(C)** Transvaginal sonography showing a few flow signals in the separates with color Doppler (arrow). **(D)** Pulsed wave Doppler demonstrating a low resistant index of blood flow in the lesion.

**Figure 3:**
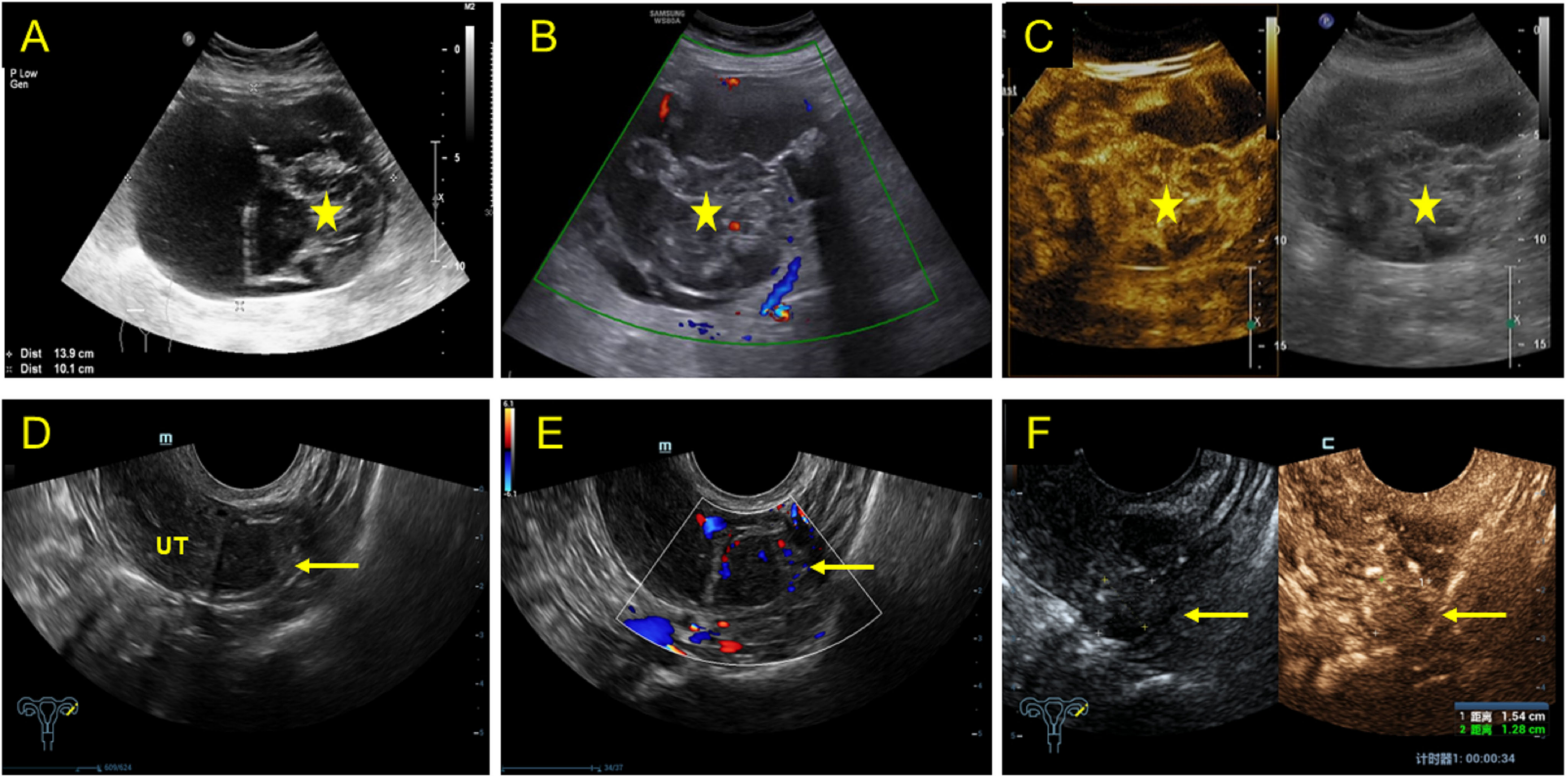
2D, CDFI, and CEUS characteristics in a mixed cystic and solid lesion (upper row) and a solid lesion (lower row). **(A)** 2D transabdominal ultrasound showing a mixed cystic and solid (asterisk) lesion in the right adnexal area with a diameter of 13.9 × 10.1 cm. **(B)** Rich blood flow was detected in the protrusions and solid part. **(C)** Transverse abdominal contrast-enhanced ultrasound showing the lesion significantly enhanced at 15 s after contrast agent injection and then slowly washed out. **(D)** Transverse transvaginal sonography showing a solid lesion (arrow) in the left ovary with a diameter of 1.70 × 1.34 cm. **(E)** Color Doppler image showing rich blood flow at the inner side of the solid lesion. **(F)** Transverse transvaginal contrast-enhanced ultrasound demonstrating that the solid lesion of the left adnexal area began showing equal enhancement at 24 s after contrast agent injection and peaked at 42 s.

**Figure 4:**
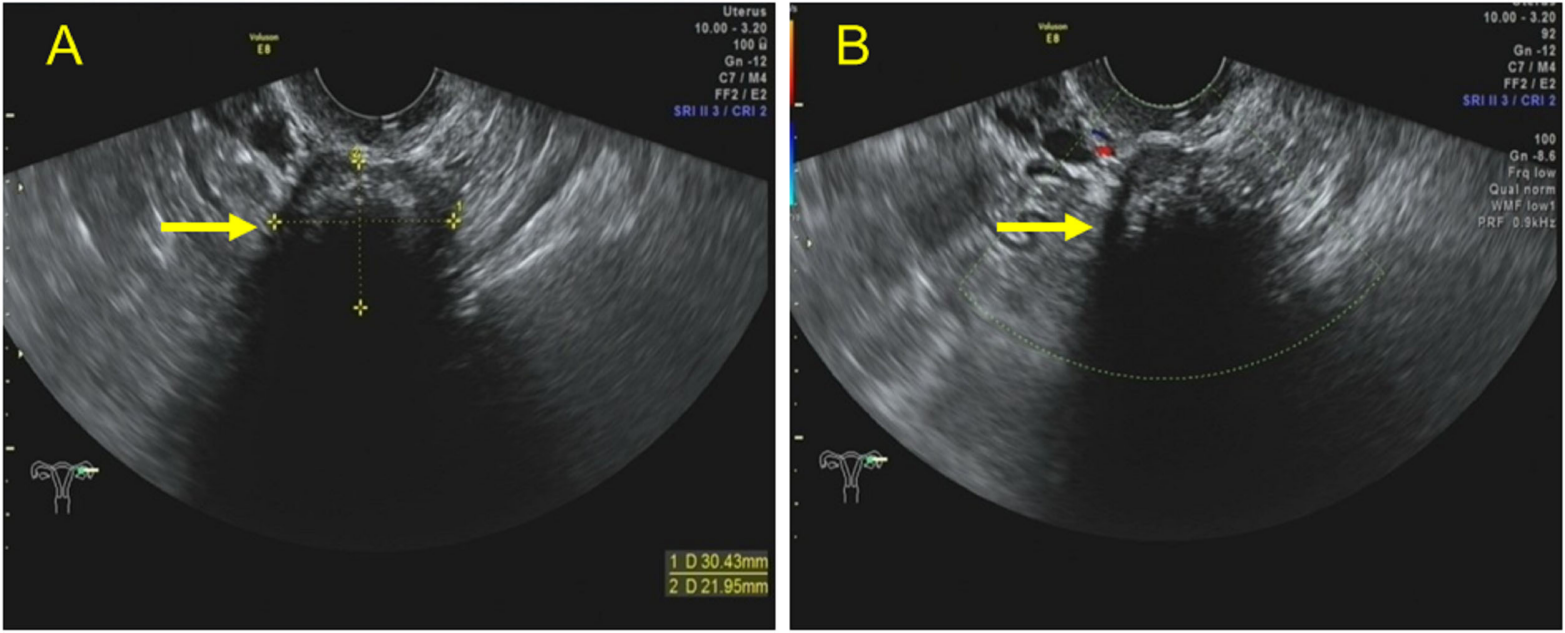
Transvaginal ultrasound images of a solid lesion with obvious calcifications in the left ovary. **(A)** Transvaginal ultrasound revealing a lesion with a diameter of 3.0 × 2.2 cm in the left ovary as well as arc-shaped calcifications in the nearer field and acoustic shadows in the rear. **(B)** Color Doppler imaging demonstrated a few flow signals in the hypoechoic part of the lesion.

**Figure 5:**
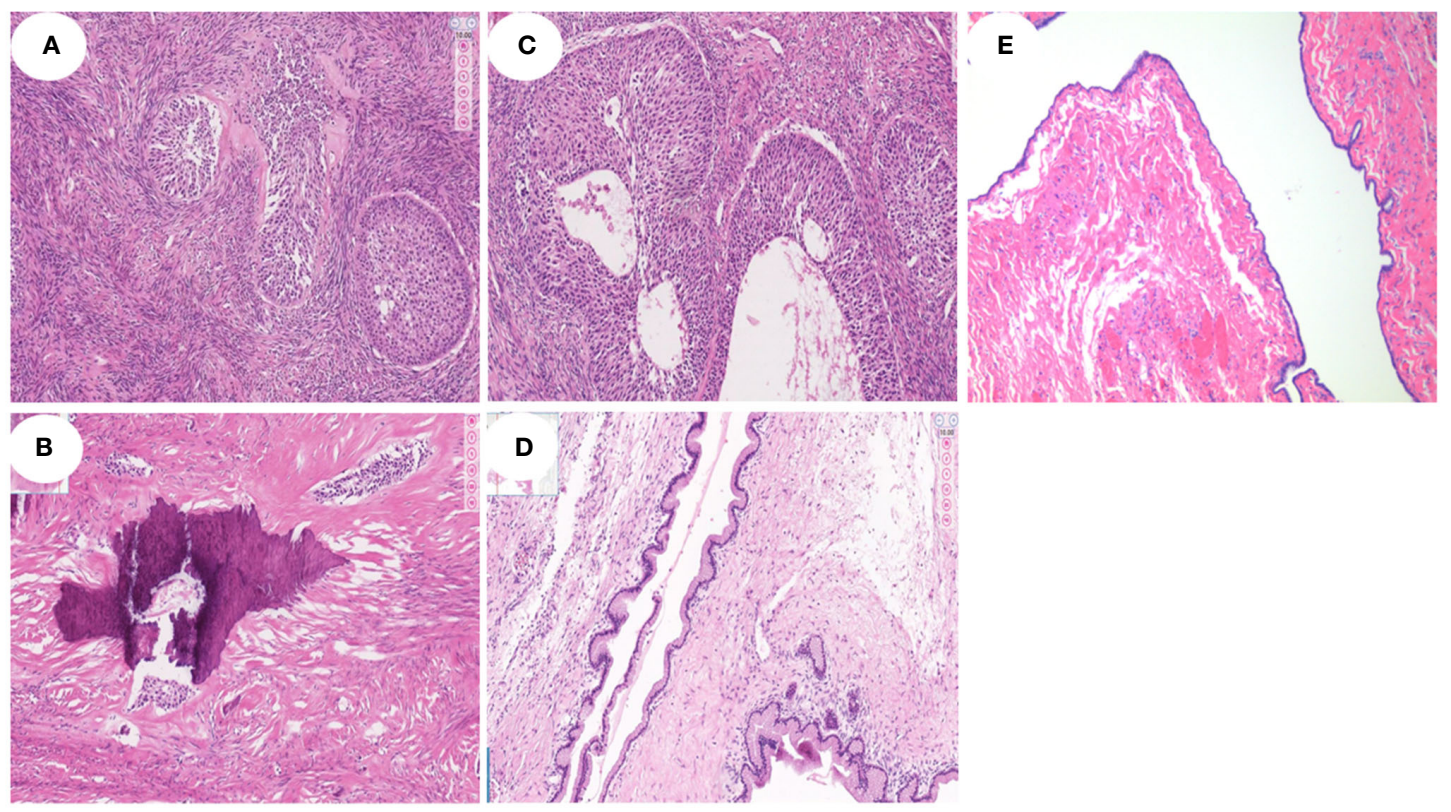
Histopathological findings of different types of BBT (HE staining, all ×100). **(A, B)**. Histopathological examination revealed one pure BBT patients, which demonstrated that nests of well-circumscribed ovoid or irregular transitional cells were seen in the dense fibrous stroma in the left ovary. And, significant calcification can be found in the tumor in figure **(B)**. **(C, D)**. Histopathological findings of a patient mixed myxoid cystadenoma component with BT, and the mucinous epithelial cells were seen in the cystic wall of tumor in the figure **(D)**. **(E)** The serous epithelial cells were seen in the cystic wall of a patient mixed serous cystadenoma component with BT.

Furthermore, 53.84% (14/26) of the lesions were mixed cystic and solid type (**Figure 2A**). The lesions were large, with light spots, and had a maximum diameter of 5.0–16.0 cm. Multiple thick hyperechoic separate and moderate echo protrusions, with irregular shapes, were also observed in these cystic lesions (**Figure 3A**). Through CDFI, rich blood flow was detected in the protrusions and solid part (**Figures 2B, C**, **3B**), and PW demonstrated a low resistant index (**Figure 2D**). In addition, 38.46% (14/26) of the lesions were solid, with a maximum diameter of 1.7-4.5 cm (**Figures 3D, E**). Calcification of different sizes could be seen in six among 14 lesions, which could be unilateral or multiple (**Figure 4A**). Through CDFI, few blood flow signals were observed in the hypoechoic part of the lesion (**Figure 4B**); (5) abdominal effusion: 4.0% (1/25) of the patients had abdominal effusion, which was myxoid cystadenoma with BT; (6) complicated uterine fibroids: 25.0% (2/8) of the patients had cystic lesions, 25.0%(1/4) of the patients had mixed cystic and solid lesions, and 46.15% (6/13) of patients had solid lesions, which complicated with uterine fibroids, as confirmed by pathology; (7) intrauterine polyps: 14.29% (1/7) of cystic type, 50.0% (2/4) of capsule solid mixed type, and 30.77% (4/13) of solid type were pathologically confirmed to be the types of endometrial polyps present (**Table 2**); (8) CEUS features: CEUS examination was performed in four of the patients. One patient had mixed cystic and solid lesion, with the contrast agent infusing at 15 s in solid part after the injection. The contrast agent filling was highly enhanced in the separated and solid parts and then slowly washed out (**Figure 3C**), which was confirmed by pathology as myxoid cystadenoma complicated with BT (**Figures 5C, D**). The others three were the solid type, which were confirmed by histopathological examination as pure BBT (**Figures 5A, B**). After injection of the contrast agent, the solid lesion in the left adnexal area began to infuse at 24 s, presenting equal enhancement. After reaching the peak value at 42 s, it simultaneously cleared with the uterus (**Figure 3F**).

**Table 2:** Two-dimensional and color Doppler ultrasound findings of the 25 patients with 26 benign Brenner tumor lesions.

Ultrasonic classification		Cystic (*n* = 8)	Capsule solid Mixed (*n* = 4)	Solid (*n* = 14)
Number		30.76% (8/26)	15.38% (4/26)	53.85% (14/26)
Position	Left	12.0% (3/25)	8.0% (2/25)	24.0% (6/25)
	Right	20.0% (5/25)	8.0% (2/25)	24.0% (6/25)
	Bilateral			4.0% (1/25)
Maximum diameter (cm)		2.1–16.0	4.0–16.0	1.7–11.0
Average diameter (cm)		5.37 ± 4.27	12.73 ± 0.78	2.27 ± 0.95
Shape	Regular	30.77% (8/26)	15.38% (4/26)	42.31% (11/26)
	Irregular	0	0	11.54% (3/26)
Border	Clear	30.77% (8/26)	11.54% (3/26)	42.31% (11/26)
	Unclear		3.84% (1/26)	11.54% (3/26)
Cystic fluid	Clear	87.5% (7/8)		
	Unclear	12.5% (1/8)	100% (4/4)	
	No			100% (14/14)
Separated	Yes	62.5% (5/8)	100% (4/4)	
	No	37.5%(3/8)		100% (14/14)
Parenchymal echo and blood flow in the sac	Yes	0	100% (4/4)	0
	No	100% (8/8)	0	100% (14/14)
Calcification	Yes		0	42.85% (6/14)
	No	100% (8/8)	100% (4/4)	57.14% (8/14)
Associated with ascites	Yes		4.0% (1/25)	
	No	32.0% (8/25)	12.0% (3/25)	52.0% (13/25)
Associated with uterine fibroids	Yes	8.0% (2/25)	4.0% (1/25)	24.0% (6/25)
	No	24.0% (6/25)	12.0% (3/25)	28.0% (7/25)
Associated with endometrial polyp	Yes	4.0% (1/25)	8.0% (2/25)	16.0% (4/25)
	No	28.0% (7/25)	8.0% (2/25)	36% (9/25)
Ultrasound diagnosis (IOTA system)	Benign	30.77% (8/26)		42.30% (11/26)
	Uncertain			11.54% (3/26)
	Malignant		15.38% (4/26)	

Values are expressed as n (%).

### Pathological characteristics

A total of 25 patients underwent surgery, with 26 ovarian lesions removed. Among them, 72.0% (18/25) were treated with total laparoscopic hysterectomy and 24.0% (6/25) with salpingo-oophorectomy laparoscopy. Only one patient was treated with total abdominal hysterectomy for the huge volume of the tumor. After the surgery, it was confirmed by pathology that 18 cases were pure BBT, three cases were BBT combined with mucinous cystadenoma, and the remaining four cases were BBT combined with serous cystadenoma (**Figure 5E**), seromucinous tumor, ovarian fibroma, and endometrioid cyst, respectively.

## Discussion

BTs are derived from the germinal epithelium with multiple differentiation potentials on the surface of the ovary, also known as ovarian fibro epithelial tumors (12). They are mainly composed of special nests of epithelial cells and tightly arranged spindle cells or fibrous connective tissues surrounding the epithelial cells (12). BT can occur at any age but is more common in people older than 50 years. It occurs unilaterally more than bilaterally (13). Its clinical symptoms are non-specific, including non-specific abdominal distension, abdominal pain, mass, and irregular vaginal bleeding in some postmenopausal women (8). Some BTs are detected incidentally during routine examinations. This study included 25 patients, of whom 23 (92.0%) were postmenopausal, six (24%) had lower abdominal pain and discomfort, and three (12%) had vaginal bleeding; the remaining patients had normal physical examination results. The cause of vaginal bleeding in BT patients was the differentiation of epithelial cells with multi-directional differentiation potential into tissue components with hormone secretion function based on cytology (12). Therefore, some juvenile patients experience estrogen-related symptoms, such as early menstruation, breast development, genital development, and sexual precocity. In adults, the most obvious symptoms are functional uterine bleeding, amenorrhea, irregular vaginal bleeding following menopause, endometrial hyperplasia, and presence of endometrial polyps and uterine fibroids. In this study, 19 patients underwent sex hormone examination, of whom two were found to have elevated estrogen levels. Of the 25 patients included in this study, seven had polyps, including one with cervical polyps and nine with uterine fibroids.

BT is a rare ovarian epithelial tumor with a low incidence (4). It can be classified into benign, borderline, and malignant, of which approximately 90% are benign and approximately 10% are borderline and malignant (4). The diagnosis and differential diagnosis of BT mainly depend on its pathology. The pathological features of BBTs are scattered epithelial nests and densely proliferating fibrous stroma with small nucleoli, broad and clear cytoplasm, squamous metaplasia in small foci, and local calcification (14). Borderline and malignant BTs are rare and have few related studies (15, 16). Histologically, BBT consists of a large number of densely proliferating fibrous stroma with various sizes of solid echoes, which are hypoechoic with posterior attenuation on ultrasound images. The local calcification in pathology, due to the different degrees of calcification, can be punctate, cluster, and patchy. Thus, the ultrasound shows various forms of hyperecho, followed by an acoustic shadow. For the detection of calcification, CT and MRI diagnosis were mostly reported in the previous literature (17). Nowadays, ultrasound can also show calcification well, and it is economical without radiation damage. In this study, 14 of 26 BBTs were of solid type, and six of them had calcifications. The diameter of the solid lesions was 1.7–11.0 cm, and the posterior echo attenuation was obvious; eggshell calcification was also observed. This is consistent with the study conducted by Moon et al. (18), who stated that most solid BTs are accompanied by an amorphous calcification and that extensive calcification within the solid component is the typical pathological manifestation of BT. This suggests that calcification is an important characteristic of BBT and that it should be considered when there is a solid lesion with calcification on ultrasound images.

As described above, BTs with solid calcification may be an important ultrasound characteristic of BBTs. On the contrary, non-calcified solid BTs are often misdiagnosed as malignant tumors due to their borders, morphology, and blood flow distribution. The solid type of tumor without calcification and ovarian malignant tumors were difficult to distinguish on ultrasound features in postmenopausal women. In this study, 14 patients were of the solid type, and eight of them were without calcification. Among the patients without calcification, three showed an irregular morphology and an unclear boundary in two-dimensional ultrasound images, and color Doppler showed the blood flow distribution. To further demonstrate the microcirculation, CEUS was performed all three patients, which indicated that the intratumoral blood supply was sparse and isoenhanced, which regressed synchronously with the uterus, and was suspected to be uncertain according to the IOTA system. The presence of sparse blood flow within the tumor in CEUS with enhancement and regression synchronizing with the myometrium was the reason for the diagnosis of uncertain rather than malignant. Therefore, CEUS played an important role in the diagnosis. However, the postoperative pathology results confirmed BBT. Of the six patients with mixed BBT on ultrasound images, three were pathologically confirmed to have mucinous cystadenoma. The ultrasonographic characteristics of these three cases were summarized as follows. The main manifestation was a large cystic echo with the attachment of solid components, and blood flow could be detected in the solid parts, which overlapped with the ultrasound characteristics of ovarian malignant tumors. One case was suspected to be malignant due to the continuous hyperenhancement of the contrast medium in the separate and solid part of CEUS, but the pathological diagnosis was also mucinous cystadenoma combined with BBT. The reason for the enhancement such as CEUS in benign Brenner tumors may be caused by the incomplete basal layer of vascular endothelial cells in the tumor. The incomplete basal layer of vascular endothelial cells causes the contrast agent to pass through the gap of endothelial cells quickly, which shows rapid regression on contrast-enhanced ultrasound images (10).

In our study, 18 (72%) cases were pure BBT in pathology, which was consistent with the literature that the vast majority of BTs were benign (19). It was also reported that about 20% of BTs could be accompanied by mucinous cystadenoma, serous cystadenoma, benign cystic teratoma, or struma ovarii (20). Similarly, in our study, seven patients (28%) had concomitant benign ovarian tumors: four cases were BBT combined with serous cystadenoma, seromucinous tumor, ovarian fibroma, and endometrioid cyst, respectively, and the other three were associated with mucinous cystadenoma, which was similar to what Roma and Masand reported (21, 22). Some scholars believe that the reason why mucinous cystadenoma and BBT can coexist is that they have a common clonal origin (23, 24). The other authors suggested that the combination of these two tumors is due to the differentiation of BBT cells (25). When the two coexist, the continuous secretion of epithelial cells in the cystadenoma leads to increased cystic fluid, which will push the BT tissue to the thinner side of the tumor. Thus, in the pathological diagnosis of large mucinous cystadenoma, careful observation of the surrounding tissues of the tumor is important to determine whether there is a small BT pathological tissue (26). The difference between BT and mucinous cystic tumors is that the latter have a single component, have no transitional epithelial nests, and are positive for the cytokeratin 20 (CK20) immunophenotype, whereas the former do not express CK20. The main basis for differential diagnosis is immunohistochemical analysis (27). However, CK20 can also be positive in patients with mucinous cystadenoma combined with BBT, and other immune markers should be combined for differential diagnosis (28).

However, the correlation between BBT and tumor markers is still unclear (29). There are studies investigating the predictive value of CA-125 level and malignancy, indicating that CA-125 alone is not suitable to differentiate between benign and malignant adnexal lesions (25). Until now, the correlation between BBT tumors and serum tumor markers, like CA-153, CA-724,and CA-199, has not been clearly concluded. These markers were elevated, which seemed more likely by chance, but the result was sometimes difficult to diagnose with ovarian malignancy, especially when the tumor showed mixed cystic and solid type or solid. Some of the mixed cystic and solid BBT tumors could be combined with cystadenoma. The association between BT and cystadenoma has also been reported by Abbas (26) in postmenopausal women. As for the increase in estrogen levels, the mechanism remains unclear. It has been reported that steroid hormones are produced by ovarian tumors probably via the following mechanisms. One is that the tumor cells themselves produce hormones, such as granular and Sertoli cell tumors. The other category is stromal cells, but not tumor cells producing hormones (30). In this study, two of the 25 patients had elevated serum estrogen levels following menopause, which may have been the cause of endometrial hyperplasia in these patients. However, whether the cause of estrogen elevation was tumor cells or stromal cells needs further study.

As for the choice of surgical methods, 72.0% (18/25) were treated with total laparoscopic hysterectomy and 24.0% (6/25) with salpingo-oophorectomy laparoscopy. Moreover, 4% (1/25) of the patients chose open surgery, which was inconsistent with the literature reports (31). The reason may be the large size of the tumor and the presence of potential malignancy. However, this procedure has a long recovery time. With the continuous development of laparoscopic technology, 96.0% (24/25) of the patients underwent laparoscopy. The surgical trauma is small, and the recovery is fast (5). Meanwhile, the surgical procedure will not cause spilling of potential malignant cells into the abdominal cavity. Laparoscopy is a very good choice for young women, especially those with fertility potential and desire. In our study, 24.0% (6/25) of the patients chose salpingo-oophorectomy laparoscopy to avoid overtreatment.

Due to the rare occurrence of malignant and borderline BTs, most of the ultrasound characteristics reported in the literature were mainly benign. It has been reported that malignant BT is usually bilateral, presenting mixed cystic and solid components or dominated by cystic components, with abundant tumor blood supply and low spectral resistance (5). However, in our study, there was only one patient with a bilateral tumor that was benign. Zheng (32) reported that borderline BTs were usually large, unilocular, or multilocular and may be cystic or capsule solid. Distinguishing BBTs is difficult sometimes and relies only on immunohistochemistry (27). Due to the limited sample size of this study, confirmation by multi-center studies is warranted.

## Conclusion

The ultrasound manifestation of BBT is variable and diagnosis challenging. Features such as calcified acoustic shadow, solid components with sparse blood flow, and prevailing of the right appendage may indicate BBT. Unilateral ovarian lesion with punctate calcification is another important feature of BBT. For adnexal solid or mixed cystic and solid masses, if the CEUS result shows isoenhancement or hyperenhancement, the possibility of BBT cannot be excluded. Contrast-enhanced ultrasound can enhance the confidence of patients with benign ovarian tumors, reduce the psychological stress of patients, and allow them to choose rational surgery. We believe that this study will improve the understanding of the clinical manifestations, laboratory examination, and ultrasound manifestations of this rare entity.

## Data availability statement

The datasets presented in this study can be found in online repositories. The names of the repository/repositories and accession number(s) can be found in the article/supplementary material.

## Ethics statement

The studies involving humans were approved by the Bioethics Committee of Ningbo First Hospital, School of Medicine, Ningbo University, China (no. 2023RS125). The studies were conducted in accordance with the local legislation and institutional requirements. Written informed consent for participation in this study was provided by the participants’ legal guardians/next of kin. Written informed consent was obtained from the individual(s) for the publication of any potentially identifiable images or data included in this article.

## Author contributions

MC: Funding acquisition, Investigation, Methodology, Writing – original draft, Writing – review & editing. SL: Formal analysis, Software, Visualization, Writing – original draft, Writing – review & editing. YC: Data curation, Supervision, Writing – original draft. MM: Formal analysis, Resources, Supervision, Writing – original draft. XJ: Data curation, Validation, Visualization, Writing – review & editing. SZ: Formal analysis, Methodology, Project administration, Software, Writing – original draft. YX: Conceptualization, Investigation, Project administration, Resources, Supervision, Writing – original draft, Writing – review & editing.

## References

[1] Lamping JD Blythe JG. Bilateral Brenner tumors: a case report and review of the literature. Hum Pathol. (1977) 8:583–5. doi: 10.1016/s0046-8177(77)80117-2 903146

[2] Seidman JD Russell P Kurman RJ. Surface epithelial cells of the ovary. In: Kurman RJ, editor. Blaustein’s Pathology of the Female genital tract. Springer-Verlag, New York (2002). p. 791–904.

[3] Meinhold-Heerlein I Fotopoulou C Harter P Kurzeder C Mustea A Wimberger P. The new WHO classification of ovarian, allopian tube, and primary peritoneal cancer and its clinical implications. Arch Gynecol Obstet. (2016) 293:695–700. doi: 10.1007/s00404-016-4073-2 26894303

[4] Kato H Kanematsu M Furui T Morishige K Hirose Y. Ovarian mucinous cystadenoma coexisting with benign Brenner tumor: MR imaging findings. Abdom Imaging. (2013) 38:412–6. doi: 10.1007/s00261-012-9887-1 22476372

[5] Nasioudis D Sisti G Holcomb K Kanninen T Witkin SS. Malignant Brenner tumors of the ovary; a population-based analysis. Gynecol Oncol. (2016) 142:44–9. doi: 10.1016/j.ygyno.2016.04.538 27130406

[6] Silverberg SG. Brenner tumor of the ovary: A clinicopathologic study of 60 tumors in 54 women. Cancer. (1971) 28:588–96. doi: 10.1002/1097-0142(197109)28:3<588::aid-cncr2820280310>3.0.co;2-j 5096924

[7] Roth LM Czernobilsky B. Ovarian brenner tumors. II. Malignant. Cancer. (1985) 56:592–601. doi: 10.1002/1097-0142(19850801)56:3<592::aid-cncr2820560328>3.0.co;2-a 4005816

[8] Yoonessi M Abell MR. Brenner tumors of the ovary. Obstet Gynecol. (1979) 54:90–6. 10.1097/00006250-197907000-00021 450368

[9] Green GE Mortele KJ Glickman JN Benson CB. Brenner tumors of the ovary: sonographic and computed tomographic imaging features. J Ultrasound Med. (2006) 25:1245e51. doi: 10.7863/jum.2006.25.10.1245 16998096

[10] Dierickx I Valentin L Van Holsbeke C Jacomen G Lissoni AA Licameli A. Imaging in gynecological disease (7): clinical and ultrasound features of Brenner tumors of the ovary. Ultrasound Obstet Gynecol. (2012) 40:706–13. doi: 10.1002/uog.11149 22407678

[11] Timmerman D Valentin L Bourne TH Collins WP Verrelst H Vergote I. Terms, definitions and measurements to describe the sonographic features of adnexal tumors: a consensus opinion from the International Ovarian Tumor Analysis (IOTA) Group. Ultrasound Obstet Gynecol. (2000) 16:500–5. doi: 10.1046/j.14690705.2000.00287.x 11169340

[12] Kuhn E Ayhan A Shih I Seidman JD Kurman RJ. Ovarian Brenner tumour: a morphologic and immunohistochemical analysis suggesting an origin from fallopian tube epithelium. Eur J Cancer. (2013) 49:3839e49. doi: 10.1016/j.ejca.2013.08.011 24012099

[13] Blaustein A. Brenner tumors. In: Pathology of the female genital tract. Springer-Verlag, New York (1982). p. 547–53.

[14] Weinberger V Minář L Felsinger M Ovesná P Bednaříková M Číhalová M. Brenner tumor of the ovary - ultrasound featuresand clinical management of a rare ovarian tumor mimicking ovarian cancer. Gineko Pol. (2018) 89:357–63. doi: 10.5603/GP.a2018.0061 30091444

[15] AkmanLAkdemirATerekMCZekiogluO. Ovarian Malignant Brenner tumor in patients over 65 years of age. Kaohsiung J Med Sci. (2014) 30:159 –160. doi: 10.1016/j.kjms.2013.01.020 24581217

[16] Albu DF Albu C Goganau AM Albu ŞD Mogoantă L Edu A. Borderline Brenner tumors associated with ovarian cyst-case presentation. Rom J Morphol Embryol. (2016) 57:893 –898. 27833989

[17] Montoriol PF Hordonneau C Boudinaud Molnar I Abrial C Kossai M. Benign Brenner tumour of the ovary: CT and MRI features. Clin Radiol. (2021) 76:593–8. doi: 10.1016/j.crad.2021.03.018 33933275

[18] Moon WJ Koh BH Kim SK Kim YS Rhim HC Cho OK. Brenner tumor of the ovary: CT and MR findings. J Comput Assist Tomogr. (2000) 24:72–76. doi: 10.1097/00004728-200001000-0001 10667663

[19] Yüksel D Kılıç C Çakır C Kimyon Cömert G Turan T Ünlübilgin E. Brenner tumors of the ovary: clinical features and outcomes in a single-center cohort. J Turk-Ger Gynecol A. (2022) 23:22–7. doi: 10.4274/jtgga.galenos.2021.2021.0001 PMC8907439 35000896

[20] Hwang CS Lee CH Lee SJ Kim YG Kim A Park DY. A peculiar case report of extraovarian Brenner tumor arising in the omentum. World J Surg Oncol. (2017) 15:72. doi: 10.1186/s12957-017-1135-2 28351362 PMC5370456

[21] Roma AA Masand RP. Different staining patterns of ovarian Brennertumor and the associated mucinous tumor. Ann Diagn Pathol. (2015) 19:29–32. doi: 10.1016/j.anndiagpath.2014.12.002 25596159

[22] Maghbool M Samizadeh B. Mixed mucinous cystadenoma with benign Brenner tumor in a huge ovarian mass, a case report and review of literature. Int J Surg Case Rep. (2022) 92:106859. doi: 10.1016/j.ijscr.2022.106859 35245850 PMC8891951

[23] Wang Y Wu RC Shwartz LE Haley L Lin MT Shih IeM. Clonality analysis of combined Brenner and mucinous tumours of the ovary reveals their monoclonal origin. J Pathol. (2015) 237:146–51. doi: 10.1002/path.4572 PMC4703556 26095692

[24] Tafe LJ Muller KE Ananda G Mitchell T Spotlow V Patterson SE. Molecular genetic analysis of ovarian Brenner tumors and associated mucinous epithelial neoplasms: high variant concordance and identification of mutually exclusive RAS driver mutations and MYC amplification [J]. Am J Pathol. (2016) 186:671–7. doi: 10.1016/j.ajpath.2015.11.008 PMC5903309 26797085

[25] Valentin L Jurkovic D Van Calster B Testa A Van Holsbeke C Bourne T. Adding a single CA 125 measurement to ultrasound imaging performed by an experienced examiner does not improve preoperative discrimination between benign and Malignant adnexal masses. Ultrasound Obstet Gynecol. (2009) 34:345–54. doi: 10.1002/uog.6415 19585547

[26] Abbas AM Amin MT. Brenner's tumor associated with ovarian mucinous cystadenoma reaching a huge size in postmenopausal woman. J Cancer Res Ther. (2015) 11:1030. doi: 10.4103/0973-1482.151858 26881612

[27] Logani S Oliva E Amin MB Folpe AL Folpe AL Young RH. Immunoprofile of ovarian tumors with putative transitional cell (urothelial) differentiation using novel urothelial markers: histogenetic and diagnostic implications. Am J Surg Pathol. (2003) 27:1434–41. doi: 10.1097/00000478-200311000-00005 14576476

[28] Seidman JD Khedmati F. Exploring the histogenesis of ovarian mucinous and transitional cell (Brenner) neoplasms and their relationship with Walthard cell nests: a study of 120 tumors. Arch Pathol Lab Med. (2008) 132:1753–60. doi: 10.1043/1543-2165-132.11.1753 18976011

[29] Buttin BM Cohn DE Herzog TJ. Meigs' syndrome with an elevated CA 125 from benign Brenner tumors. Obstet Gynecol. (2001) 98:980–2. doi: 10.1016/s0029-7844(01)01562-9 11704231

[30] Hiroi H Osuga Y Tarumoto Y. A case of estrogen-producing Brenner tumor with a stromal component as a potential source for estrogen. Oncology-Basel. (2002) 63:201–4. doi: 10.1159/000063810 12239457

[31] Salibay CJ Zanfagnin V Miller H Wang T Brunette LL Wang T. Borderline brenner tumor of the ovary coexisting with an ovarian mucinous cystadenoma with focal atypical epithelial proliferation: a rare case with review of the literature. Int J Surg Pathol. (2021) 29(7):788–93. doi: 10.1177/1066896921999459 33635096

[32] Zheng Heller DS . Borderline brenner tumor: A review of the literature. Arch Pathol Lab Med. (2019) 143:1278–80. doi: 10.5858/arpa.2018-0285-RS 30779594

